# TRAVeLer: a tool for template-based RNA secondary structure visualization

**DOI:** 10.1186/s12859-017-1885-4

**Published:** 2017-11-15

**Authors:** Richard Elias, David Hoksza

**Affiliations:** Faculty of Mathematics and Physics, Charles University, Prague, 11800 Czech Republic

**Keywords:** Visualization, RNA secondary structure, Template-based modeling, Software tool

## Abstract

**Background:**

Visualization of RNA secondary structures is a complex task, and, especially in the case of large RNA structures where the expected layout is largely habitual, the existing visualization tools often fail to produce suitable visualizations. This led us to the idea to use existing layouts as templates for the visualization of new RNAs similarly to how templates are used in homology-based structure prediction.

**Results:**

This article introduces Traveler, a software tool enabling visualization of a target RNA secondary structure using an existing layout of a sufficiently similar RNA structure as a template. Traveler is based on an algorithm which converts the target and template structures into corresponding tree representations and utilizes tree edit distance coupled with layout modification operations to transform the template layout into the target one. Traveler thus accepts a pair of secondary structures and a template layout and outputs a layout for the target structure.

**Conclusions:**

Traveler is a command-line open source tool able to quickly generate layouts for even the largest RNA structures in the presence of a sufficiently similar layout. It is available at http://github.com/davidhoksza/traveler.

**Electronic supplementary material:**

The online version of this article (doi:10.1186/s12859-017-1885-4) contains supplementary material, which is available to authorized users.

## Background

The ability to visually inspect the secondary structure of an RNA molecule is an important aspect of RNA analysis, especially in case of large molecules,such as ribosomal RNAs (rRNAs). For such molecules, suitable visualization can help to determine conserved regions shared across species or, alternatively, expansion segments, the exposed parts of the RNA structure. The visualization also facilitates the comparison of secondary structures, identification of function of RNA molecules and modeling of functional mechanisms.

There are three possible approaches with regard to laying out RNA: a linked graph, a circular graph, and a classical structure [1]. In the linked graph, the nucleotides are drawn on a straight line in sequence order, and base-paired residues are linked by an arc. The circular graph is similar to the linked graph representation with the nucleotides laying, however, on a circumference of a circle and connected with straight lines. Both of these representations lack the ability to capture the secondary structure motifs and therefore the classical structure is used when detailed visual analysis of secondary structure motifs and their interaction are needed. In the classical structure the positions of nucleotides is chosen so that the secondary structure motifs, such as hairpins, bulges, or multibranch loops can be discerned.

Since the secondary structure of RNA can be presented as a graph, the RNA visualization task can be translated to a graph drawing problem. However, there are specifics to the RNA secondary structure which do not enable the application of the general graph drawing solutions. The RNA specifics require the lengths of the edges that correspond to base pairs to be constrained, or the secondary structure motifs to be drawn in a compact and specific way. For example, hairpins should consist of a stem and a loop where stem-related nucleotides commonly lie on a line, while loop residues are located on a circle, and the resulting layout should be planar [2]. These rules maybe applied as, relatively vague, optimality criteria if needed and could drive the visualization of small RNA structures. However, there are no such rules with respect to how various secondary structure motifs should be positioned with respect to each other or how complex motifs, such as multibranch loops, should be laid out. Therefore, there is basically an infinitely many possibilities how to lay out the secondary structure of more complex RNA molecules.

The absence of rigid criteria when assessing the quality of a layout leads to the fact that secondary structure visualization is largely habitual and while the layout of small secondary structure motifs, such as hairpins, are similar in different tools, their mutual positions differ greatly among the existing visualization tools. A great and exhausting overview of secondary structure drawing approaches and software tools (both command line and interactive) can be found in a recent review by Ponty et al. [3]. The most commonly used tools for the visualization of secondary structure of RNA molecules include VARNA [4] and RNAplot [5].

**Fig. 1:**
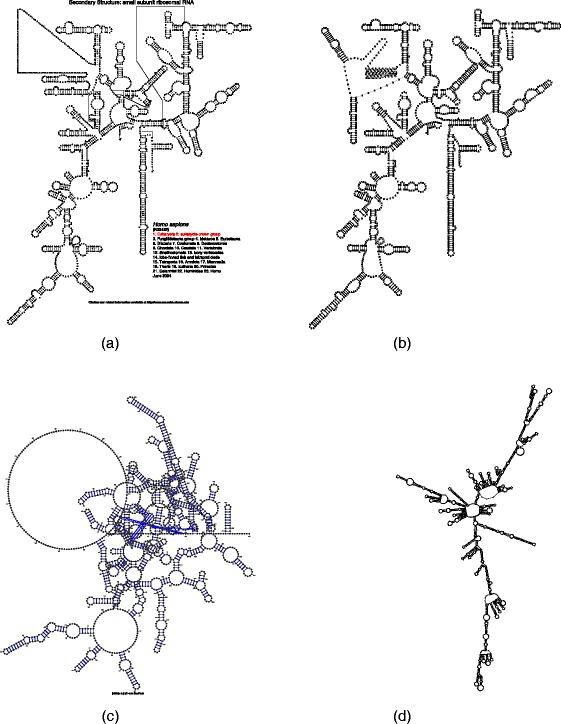
Layout of small subunit of human ribosomal RNA (GenBank accession number K03432) by different tools. The input structure definition (sequence and structure in the dot-bracket notation) can be obtained from https://github.com/davidhoksza/traveler (the *data* directory). **a** Layout in the form biological community is used to (downloaded from the CRW website [1]). **b** Layout generated by Traveler using fruit fly as a template. **c** Layout generated by VARNA (version 3-93). **d** Layout generated by RNAplot

Outputs of these tools can differ substantially which is especially true for large RNA structures. We show this on an example of the visualization of the small subunit of human rRNA which we contrast with the dramatically different layout used by the biologically community. See Fig. 1 a for the visualization of small subunit of human ribosomal RNA (GenBank accession number K03432) in the layout which biological users are used to seeing (downloaded from the Comparative RNA Website - http://www.rna.icmb.utexas.edu/). As a contrast, we show the layouts generated by Traveler, the tool introduced in this paper, VARNA, RNAplot, jViz.Rna [1] and RNAFdl [6] tools^1^.

The poorly defined optimality criteria for the secondary structure visualization motivated us to circumvent the problem by developing a template-based drawing algorithm [7] which requires on its input the secondary structure of a template RNA together with its layout and the secondary structure of the target RNA molecule for which the layout is to be generated. Then, using tree edit distance, the template layout is turned into the target one.

It should be noted that our approach is not the first one to use a template to draw an RNA secondary structure. The tool RnaViz [8, 9] allows a user to pass a so-called skeleton, which is then used when drawing target RNA^2^. To obtain the skeleton, one needs to use de novo layouting capabilities or RnaViz, and correct the overlaps manually. The resulting layout then can be stored as a skeleton and used for the visualization of other similar structure. Our approach, on the other hand, uses the template structure directly and its visualization provided either as a VARNA or CRW file (see “Traveler” section).

In this paper, we introduce a software tool called TRAVeLer (Template-based RnA VisuaLization) by implementing an extended and optimized version of our template-based drawing algorithm. The extension includes the implementation of a more efficient two-step tree edit distance (“Target-template structure matching” section), special treatment of multibranch loops (“Multibranch modification” section) a range of additional polishing steps and special cases treatments (“Postprocessing and special cases treatment” section), and the ability to use VARNA layouts as templates for visualization (“Traveler” section). Traveler is capable of visualizing even the biggest structures with thousands of nucleotides in tens of seconds; it is provided as an extendible, open-source software framework and can be downloaded from https://github.com/davidhoksza/traveler.

## Implementation

**Fig. 2:**
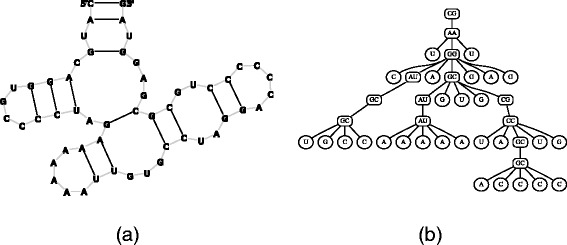
Tree-based RNA representation. Example of a secondary structure (**a**) and its corresponding tree-based representation (**b**)

The algorithm implemented in Traveler is based on the ability to represent a pseudoknot-free RNA secondary structure as an ordered rooted tree^3^. In the tree, inner nodes represent base pairs and unpaired nucleotides form leaves of the tree as illustrated in Fig. 2. To build such a tree from an input structure, one simply traverses the secondary structure in sequence-order from both ends simultaneously and transforms the encountered paired and unpaired nucleotides into inner nodes or leaves of the nascent tree. The order of neighboring nodes is defined by the order in which the nodes are encountered in the traversal.

### Target-template structure matching

Firstly, Traveler converts the target and template structures into their corresponding tree representations. In the ideal case of Fig. 2, the structure can be directly converted into a rooted tree. However, if the first and last nucleotides are not paired, an artificial root needs to be installed, otherwise the structure would be translated into a forest as is the case with most larger structure (see Fig. 1 for an example).

Secondly, tree edit distance (TED) is used to obtain mapping between the trees. TED, next to the number representing dissimilarity of the input trees, generates a minimal sequence of tree edit operations (insert, update, delete) which turns the template tree into the target one. The original TED algorithm [10] has time complexity $\mathcal {O}\left (m^{3}n^{3}\right)$, for trees with *m* and *n* nodes respectively, and memory complexity $\mathcal {O}(mn)$ which can be problematic with large structures such as ribosomal RNAs which contain several thousand nucleotides. The time complexity of original TED was improved to $\mathcal {O}\left (m^{2}n^{2}\right)$ by Zhang and Shasha [11] who introduced a special type of tree decomposition (operation needed in TED) which, when used, allows to skip some computation in the TED recursion. Another decomposition approach comes from Demain et al. [12] resulting in time complexity $\mathcal {O}\left (m^{2} n \log n\right)$. In Traveler, we have implemented a method called RTED (Robust algorithm for the TED) described in [13]. RTED allows to determine optimal decomposition for given tree resulting in a generalized version of the TED algorithm with $\mathcal {O}\left (m^{3}\right)$ worst-case time and $\mathcal {O}(mn)$ memory complexity.

### Layout transformation

TED procedure results in a mapping that is subsequently used to convert the input template layout into the target layout. Since the mapping consists of a sequence of tree edit operations, each tree edit operation (update, insert, delete) can be assigned its visual counterpart. We thus obtain a recipe how to transform the template layout into the target one. A deleting operation therefore leads to removal of a base(pair) from the template which, in turn, results into free space so the layout needs to be modified accordingly to remove the space. Analogously, insertion results in a new base(pair) and the layout needs to be shifted to accommodate the new element. Finally, an update operation does not lead to any structural layout modifications. Irrespective of the modification operation, we want to interfere with the template layout as little as possible and make only local changes of the template. This is achieved using two methods (used in both insert and remove operations) which handle the distribution of the bases over a circle (Algorithm 1) and shift a subtree in given direction (Algorithm 2).





In the following section, we discuss how the layout modification operations are handled in more detail. For more examples illustrating individual cases see the Additional file 1.

#### Inserting nodes

**Fig. 3:**
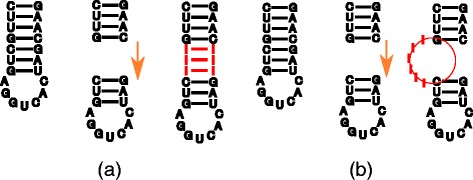
Simple modification operations. Illustration of layout modification enforced by inner (**a**) and leaf (**b**) nodes insertions

First, let us consider insertions which do not involve multibranch loops. When inserting a node, we need to discriminate between inserting an inner node and inserting a leaf node. In the first case, the operation corresponds to inserting a base pair into a stem and is handled by Algorithm 2. We insert the base pair at a given position in the layout and then shift all the nodes corresponding to the descendants of the new parent node. The direction is determined by a direction vector given by the new parent and grandparent of the inserted node (see Fig. 3). In the latter case, when a new leaf node is inserted, we need to distinguish between an insertion into an existing loop and an insertion into a stem where it forms a new bulge. Inserting into an existing loop requires redrawing the loop using Algorithm 1. One thus needs to extend the circle on which all the sibling leaves reside, i.e. the repositioning of bases corresponding to nodes comprising of siblings of the node being inserted. When inserting a leaf into a stem, i.e. a linear path in the tree, and thus forming a new bulge, is slightly more complicated since it requires shifting the tree rooted in the sibling of the newly inserted node to create space for the newly formed bulge and then position the node in the bulge the same way as when inserting into a loop. This situation is illustrated in Fig. 3 b).

Several issues can arise when inserting nodes in the first level of the tree. Such situation is discussed in “Postprocessing and special cases treatment” section.

#### Removing nodes

Removing nodes from the tree and respective layout modifications are done essentially the same way as insertions are done. The only difference is in the direction of a shift when removing a base pair from a stem and in decreasing the loop size instead of increasing it when removing a base from the loop.

#### Multibranch modification

**Fig. 4:**
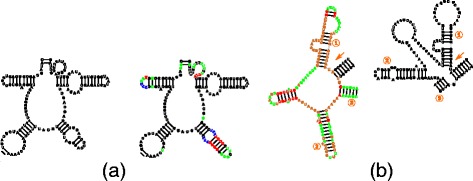
Multibranch modification (see Additional file 1 for color coding definition). **a** Multibranch modification without loop rebuild. On the left is part of frog (X04025) 18S rRNA template and on the right is the target (human 18S rRNA) visualization with the residues in the upper right part being squeezed to avoid re-layouting of the loop. **b** Multibranch modification with loop rebuild. On the left is part of shrimp (X04025) 18S rRNA template and on the right is the target (human 18S rRNA) visualization where the loop had to be rebuilt due to substantial difference of the target and template. The numbers representing the corresponding hairpins in the respective structures

In terms of the tree representation, multibranches correspond to nodes which have at least two non-leaf children. In cases of large RNA structures, the secondary structure visualizations are manually modified to be as compact as possible which results in not respecting all rules, such as the circular shape of a multibranch structure. For this reason, we try to interfere with multibranches as little as possible and treat them in a special way. Clearly, after any insertion into a multibranch loop, we could use Algorithm 1 to distribute all the base and basepairs comprising the loop. However, this would likely result in substantial modification of the layout, especially for a big loop in the center of the structure. Therefore, in situations when only few bases are added or removed, we try to squeeze or expand the bases between the respective neighboring branches to utilize the space between the branches without the need to reposition them. If this is not possible, we need to rebuild the whole loop, which requires finding positions on a circle as it is in case of simple loops. Then, we need to rotate each of the branches rooted in the modified loop. The rotation needs to be propagated into the descendants of each of the branches. Both situations are illustrated in Fig. 4.

#### Postprocessing and special cases treatment

Although we try to touch the template visualization as little as possible, after the target layout is generated we apply several modifications to the resulting layout to improve its quality.

**Fig. 5:**
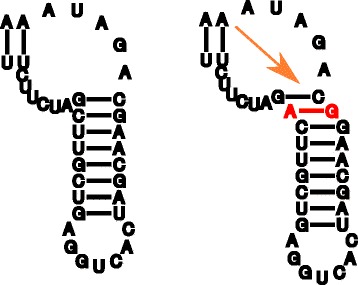
Issues when inserting a base pair. Example of incorrect position of base pair

Firstly, we straighten stem residues so that they lie on a line. It is necessary, because, for example, when inserting a base pair, the direction vector is given by the positions of the parent and grandparent, but that can lead to a curved stem as shown in Fig. 5.

Our proposed approach always arrives to a target layout, (2D) steric clashes can, however, occur in the target. This is especially true when the target and template structures are too dissimilar. Since the human-generated layouts tend to be compact and able to utilize the available space well, insertions can cause two subtrees that are adjacent in the template visualization clash in the target. To minimize the number of clashes in the target layout, we evaluate every subtree whose nodes clash with other parts of the tree and try to rotate it. We try to do several rotations and pick the one with the lowest number of clashes.

The second level of the tree requires special attention if the RNA structure is not rooted, i.e. it does not start with a base pair. This can occur quite frequently with real structures. For example, in Fig. 1 every base or base pair which is not descendant of a base pair is in the second level and their parent is the artificial root. In Fig. 1, the second level starts (from the 5’ end) with U,A,C,CG,U,AU,…. All these residues do not have a parent with a well-defined position and thus their removal would not modify the final layout. For example, by removing the first A one will end up with a space as it is not part of any loop or bulge which would be affected by its removal. Therefore, in the postprocessing phase we try to normalize the positions of the nodes in the first level with respect to each other.

Another issue is when inserting a base pair into the second level because in such a case, we cannot use parent and grandparent to correctly determine its position as there are no ancestors. In such a situation we discriminate between two cases. In the first case we insert a base pair into an existing stem, i.e. the target and template both have a branch at a given position. Then we can use the information about the position of the start of the stem from the template, use it as the position for the inserted base pair and shift the rest of the stem. In the second case, we insert a base pair which is the root of a new branch. In such a case we cannot use the position of an existing branch and we also do not have the position of a parent to guide the insertion. Therefore, we use direct siblings of the inserted branch and orient the branch perpendicular to them. Moreover, we then have to shift all the siblings to the right or left of the inserted branch.

### Traveler

The above described approach has been implemented into a software tool called Traveler. The architecture of Traveler is divided into three parts: (i) parser, (ii) mapper and (iii) visualizer.

The purpose of the parser is first, to take the target and template and generate their respective tree representations and second, to take the template layout and extract elements corresponding to bases and their interactions. The supported format of the secondary structures is the Vienna/DBN format, commonly used for RNA secondary structure representation. As for the template layout, we support two formats. Since the idea of template-based drawing is useful primarily for large structures and was developed with the intention of visualizing ribosomal RNA structures, Traveler implements image parser for postscripts visualization from the CRW database [14]. The CRW database hosts visualizations of rRNA secondary structures in the form they are used by the biological community, enabling easy, comparative visual analysis of large structures. The second input template layout format which Traveler supports is the SVG format output by VARNA. Since VARNA is a complex tool supporting various RNA visualization styles, Traveler currently supports only the simple base pairs types. However, the architecture of the application allows one easily implement a parser for a new image format and use it in Traveler. All the visualizations in the supplementary which illustrate the layout modification operations have been generated from a VARNA template layout. All the target layouts have been thus generated by Traveler using the VARNA parser.

Mapper is the core component of the application implementing the tree edit distance and corresponding layout modification operations. It is separated from the subsequent visualization and can be run independently for the user to be able to do the mapping and then visualize the mapping repeatedly with different options.

The final component of Traveler is the visualizer. Visualizer stores the resulting layout in SVG and PS formats, i.e. formats which allow simple modification of the result in any vector graphics editor. If the input template is in the VARNA format then, since the output SVG complies with VARNA, the output can be reused as a template. Similarly, one can reuse the PS output as an input template if the input format is CRW. Furthermore, the templates can be modified manually provided that the modified files comply with the structure of the CRW files (in case of PS) or VARNA files (in case of SVG). The user can also choose to color code the resulting structure so that updated, inserted and shifted residues are easy to spot. The visualization can thus be used to see where the input molecules differ with respect to their secondary structures. If the target and template structures are too dissimilar, substantial changes in the layout are required which might cause steric clashes. Therefore a switch which instructs Traveler to output the number of such overlaps and highlight them in the resulting image can be turned on. An overlap is defined as an intersection of two lines joining two pairs of residues (hydrogen bond or sugar-phosphate backbone).

## Results and discussion

**Fig. 6:**
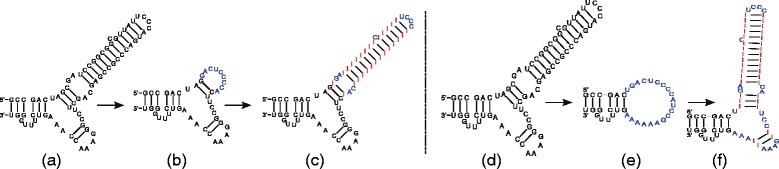
Traveler’s ability to recreate layouts. On the left hand side, we took a structure with two hairpins (**a**), removed part of a stem and used the original structure as the template (**b**). Then we reinserted the residues and used (**b**) as a template to obtain (**c**). Similarly, (**d**), (**e**) and (**f**) show re-creation of the starting structure with a more drastic middle step where the two hairpins loose residues so that the remaining residues form a loop. **f** demonstrates that Traveler is able to successfully recreate the original structure. For the purpose of clarity, the new residues were labeled I and shown in red, while the residues which needed to be repositioned are shown in blue

To illustrate the ability of Traveler to achieve the required results we have carried out several experiments. In the first experiment, we prepared an artificial RNA secondary structure and a layout, and then formed a target structure where one of the template stems was shortened, and generated its layout Fig. 6 b. Subsequently we switched the role of the template and the target which correctly resulted in a layout similar to the original template Fig. 6 c. In Fig. 6 d-f we repeated the same process but with more substantial modifications. Here, the recreated template layout slightly differs from the original one which was expected since Traveler had to rebuild the multibranch loop and its rules for positioning branches on a loop are different from the ones used to generate the original layout.

A legitimate question is how close the secondary structures of a target and template need to be for Traveler to give satisfying results. In order to quantify this, we downloaded all 16 available 18S rRNA structures from the metazoa kingdom (multicellular animals) from CRW, and generated a layout for each of the structures using every other structure from the set as the template. For each structure we thus obtained 15 tree edit distances and corresponding visualizations. For each structure the templates were sorted based on decreasing TED, and Table 1 shows the average tree edit distance and the average number of overlaps including standard deviation for each ranking. We can observe that for high tree edit distances the number of overlaps grows up to about 40 overlaps per image. For smaller distances, there is not a clear trend, but that can be ascribed to the large standard deviations in the number of overlaps (see Additional file 2 for the individual results and projects repository for the files used to generate the results).

**Table 1:** Tree edit distance and the number of overlaps when using k-th most similar structure as a template

Ranking	TED	avg(stddev)
1	85.94	6.31 (7.60)
2	113.94	12.81 (19.08)
3	137.38	11.62 (19.58)
4	158.38	8.06 (12.92)
5	172.00	4.12 (7.59)
6	189.12	7.06 (12.68)
7	196.31	4.50 (7.62)
8	199.44	4.06 (6.73)
9	258.31	26.75 (23.06)
10	262.88	22.75 (23.27)
11	265.25	8.62 (15.74)
12	271.12	27.88 (27.07)
13	301.19	12.62 (8.28)
14	345.25	33.81 (17.30)
15	805.62	44.06 (18.92)

Computed over all pairs of 18S rRNA structures from the metazoa kingdom available in CRW

**Fig. 7:**
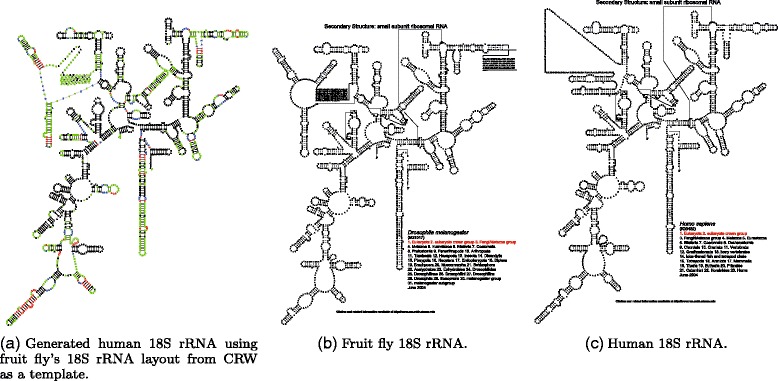
Visualization of human 18S rRNA with Traveler. **a** shows the target layout, (**b**) is the template layout while (**c**) is the desired layout as stored in the CRW. The Traveler’s output is colored so that red represent inserted residues, green are relabeled residues and blue are residues that needed to be shifted due to indels happening within given hairpin (see Additional file 1 for full color coding definition)

Having few overlaps in such a large structure as rRNA is not an issue as illustrated in Fig. 7 where we used Traveler to generate the layout for human 18S rRNA using fruit fly’s 18S rRNA as a template. The example demonstrates that even when such a relatively distant template is used the resulting layout (Fig. 7 a) is reasonable when compared to the correct layout (Fig. 7 c). The only problematic part seems to be the layout of a poorly characterized region (expansion segment) in the upper left corner of the visualization. We can see that in the template and correct target layout (Fig. 7 b and c), this region and the neighboring hairpins are laid out differently. Since the target layout is based on a template and not a target, which is not known in the time of prediction, the resulting layout resembles the template not the target. Moreover, since the long stretch of uncharacterized (unpaired) nucleotides in a template is laid out in an ad-hoc fashion, indels in this region result in mistakes in the target layout because Traveler is able to work with well-defined, hairpin-like structures only. The runtime needed to generate this layout was about 1 min on commodity hardware.

**Fig. 8:**
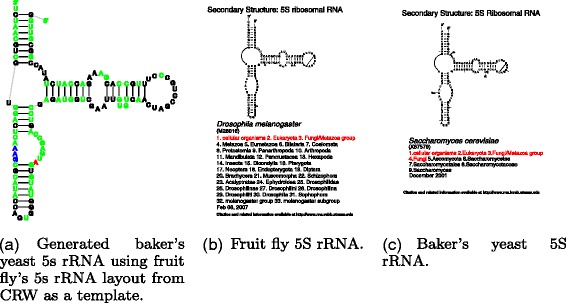
Visualization of baker’s yeast 5S rRNA with Traveler. **a** shows the target layout, (**b**) is the template layout while (**c**) is the desired layout as stored in the CRW. The Traveler’s output is colored so that red represent inserted residues, green are relabeled residues and blue are residues that needed to be shifted due to indels happening within given hairpin (see Additional file 1 for full color coding definition)

Traveler can find utilization not only as a tool for single molecule visualization, but also as a backend in any application where automatic layout of one or more RNA molecules is required. However, its low runtime makes it exceptionally suitable for large scale generation of RNA layouts for RNA types where a consensus for secondary structure layout exists. As far as we are aware, currently a strong consensus exists only for ribosomal RNAs. We have shown examples of its application to large rRNAs, but it can be equally well used for small rRNAs such as 5S rRNA, templates of which can be also found in CRW (see Fig. 8).

Although the main application we had in mind when developing Traveler was visualization or large rRNA molecules, any field of RNA research where consistent systematic layout of secondary structure is needed can benefit from utilization of a template-based layout tool such as Traveler. For example, tRNA molecules are commonly visualized with similar layout in the same orientation (5’ and 3’ ends up), so here Traveler could be used to generate standardized layout for all tRNA molecules with available secondary structure. Therefore, we also envision application of Traveler as an enabler of standardization of layouts for different RNA stubtypes. These subtypes need to share common secondary structure core so that they can benefit from application of a template-based layouting algorithm. The number of available (long) noncoding RNA secondary structures (either predicted or experimentally determined) in databases such as LNCipedia [15] (almost 150.000 structures by October 2017) indicates the potential of such application.

Finally, Traveler can be used in secondary structure prediction efforts when multiple predictions of the same sequence need to be visualized in a consistent manner to enable visual analysis of differentially predicted regions.

## Conclusions

This paper has introduced Traveler a tool capable to generate RNA secondary structure layouts which conform to biologists intuition when a template layout exists. Although it can be used for structures of any size, its major application is in visualizing large RNA structures with the focus on ribosomal RNAs where de novo tools are not capable of arriving at the expected layout and manual visualization is highly impractical.

Traveler is a command line application with no prerequisites and is freely available at http://github.com/davidhoksza/traveler.

## Availability and requirements

**Project name:** TRAVeLer

**Project home page:** https://github.com/davidhoksza/traveler

**Operating systems:** Unix/Linux

**Programming language:** C++

**License:** GNU GPL

## Endnotes

^1^ A commonly cited tool Pseudoviewer3 is not included here since we were not able to get any visualization with Pseudoviewer for the input structure.

^2^ Details on how exactly this is done are missing in both RnaViz publications.

^3^ Traveler also accepts pseudoknotted structures. Those are, however, first converted into pseudoknot-free structures and only then processed. However, the template layout can include lines corresponding to pseudoknots and these do get copied over to the target layout.

## Additional files

Additional file 1 Traveler operations. Illustration of simple insertion and deletion operations on both layout and tree level. (PDF 277 kb)

Additional file 2 Results on Metazoa 23S rRNA. Tree edit distance, number of overlaps and runtimes for all Metazoa 23s rRNA structures available in CRW. (TXT 10 kb)

## Abbreviations

CRW: Comparative RNA web site
PS: Postscript
SVG: Scalable vector graphics
TED: Tree edit distance
