# Supplementary material for: TRAVeLer: a tool for template-based RNA secondary structure visualization
Source: BMC Bioinformatics. 2017 Nov 15;18:487. doi: 10.1186/s12859-017-1885-4 (PMC5688744; doi:10.1186/s12859-017-1885-4)
Supplement: Supplementary file 1 — Traveler operations. Illustration of simple insertion and deletion operations on both layout and tree level. (PDF 277 kb) [file 12859_2017_1885_MOESM1_ESM.pdf]

Supplementary information: Traveler's layout modification operations

Traveler uses tree-based representation and tree edit distance algorithm to turn a template layout into the target one. In order to do so, it couples tree edit operations with layout modification operations. Here we show examples of simple conversions on both tree and layout level.

The color coding of nucleotides is follows:

- Red - inserted bases
- Green - edited bases - e.g. the template has an adenosine at a position while the target has a cytosine at the same position and therefore cytosine will be colored green in the resulting layout
- Blue - reinserted bases - happens when traveler needs to redraw simple structures like hairpins (for example due to the change in the number of bases)
- Brown - rotated parts - similar situation to reinserted bases, but takes place when redrawing a multibranch loop (in that case all branches need to be rotated to lie on a circle)

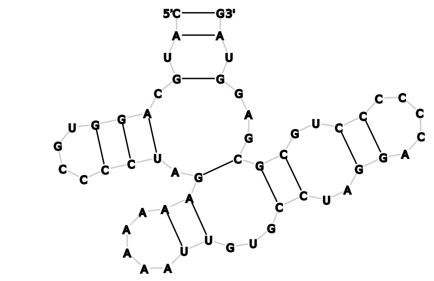

(a) Original RNA molecule

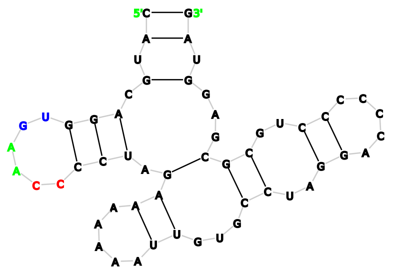

(b) Modified RNA molecule

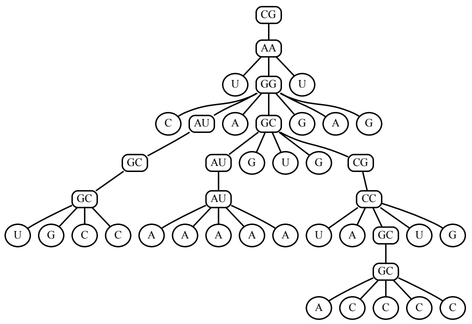

(c) Tree representation of the secondary structure of the original RNA molecule.

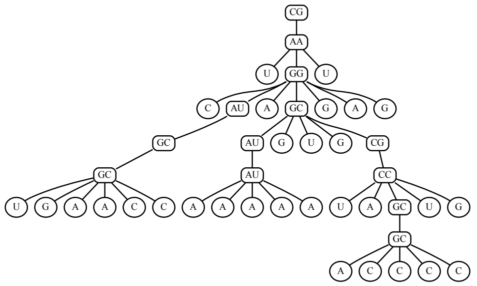

(d) Tree representation of the secondary structure of the modified RNA molecule.

Figure 1: Adding two residues to a loop.

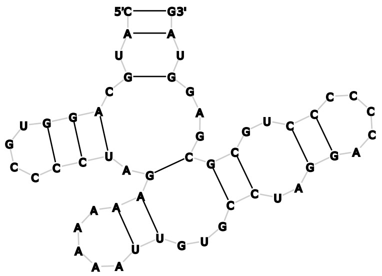

(a) Original RNA molecule

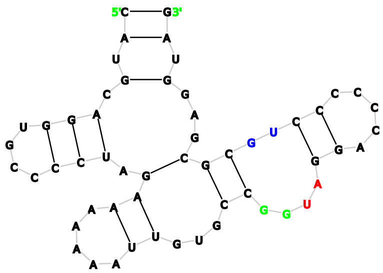

(b) Modified RNA molecule

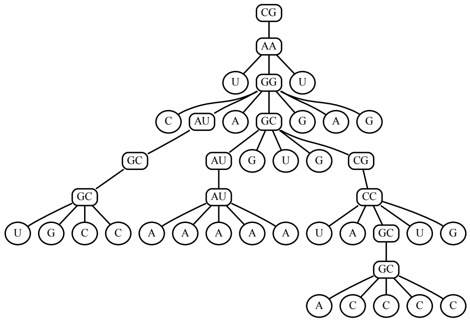

(c) Tree representation of the secondary structure of the original RNA molecule.

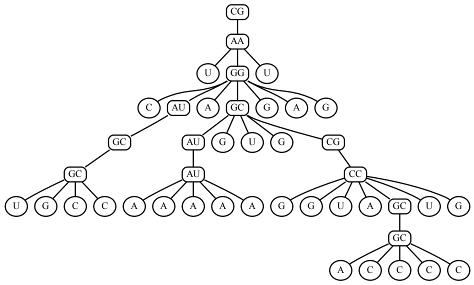

(d) Tree representation of the secondary structure of the modified RNA molecule.

Figure 2: Adding two residues to an inner loop.

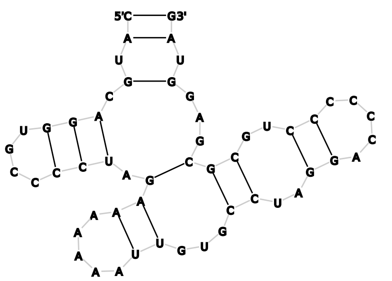

(a) Original RNA molecule

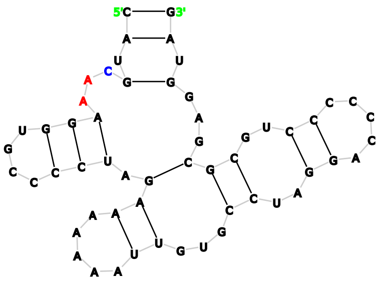

(b) Modified RNA molecule

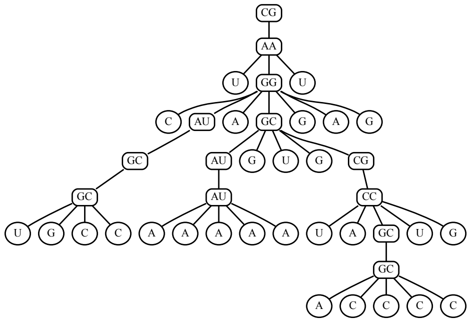

(c) Tree representation of the secondary structure of the original RNA molecule.

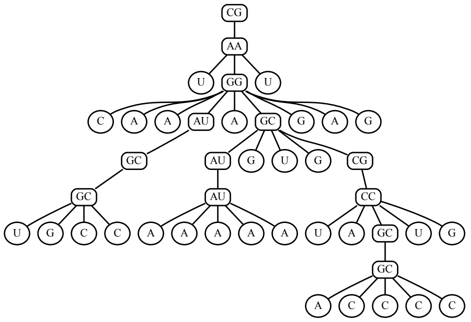

(d) Tree representation of the secondary structure of the modified RNA molecule.

Figure 3: Adding two residues to a multi-branch loop.

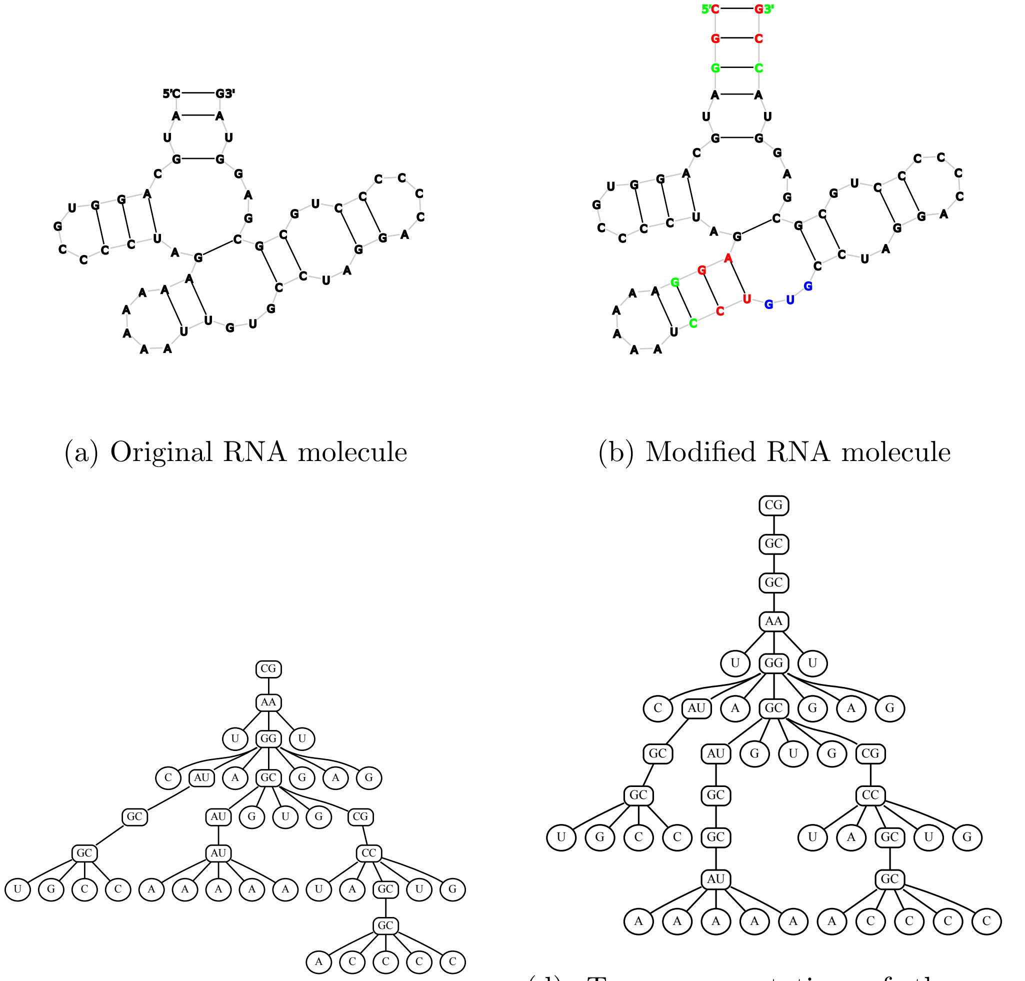

(a) Original RNA molecule

(b) Modified RNA molecule

(c) Tree representation of the secondary structure of the original RNA molecule.

(d) Tree representation of the secondary structure of the modified RNA molecule.

Figure 4: Adding two basepairs to two different hairpins.

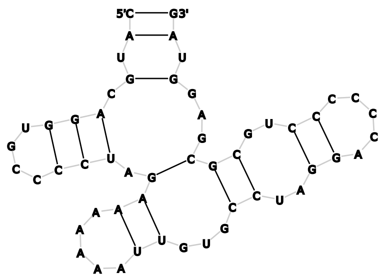

(a) Original RNA molecule

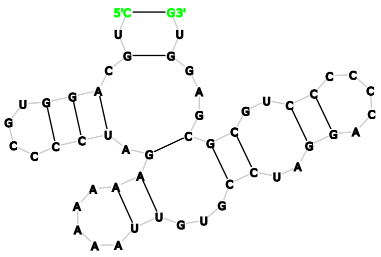

(b) Modified RNA molecule

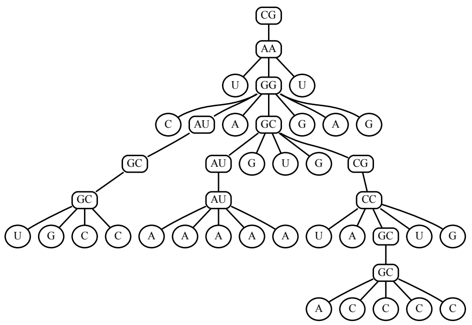

(c) Tree representation of the secondary structure of the original RNA molecule.

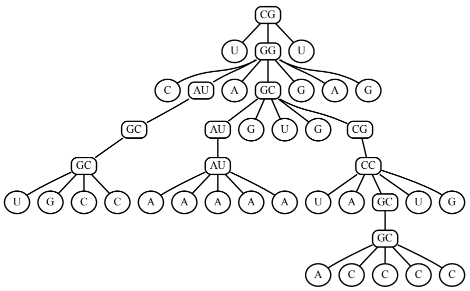

(d) Tree representation of the secondary structure of the modified RNA molecule.

Figure 5: Removing a basepair from a stem.

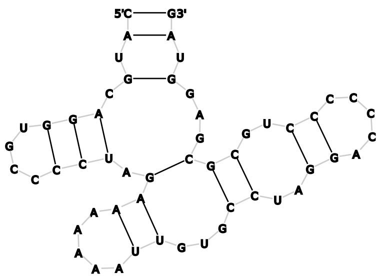

(a) Original RNA molecule

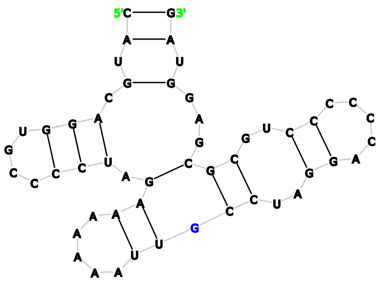

(b) Modified RNA molecule

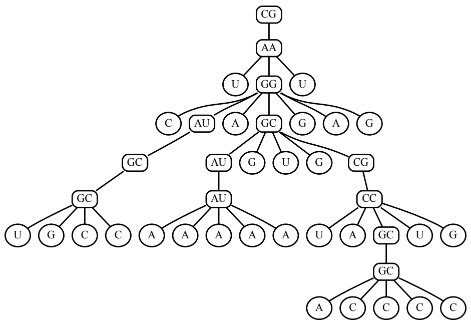

(c) Tree representation of the secondary structure of the original RNA molecule.

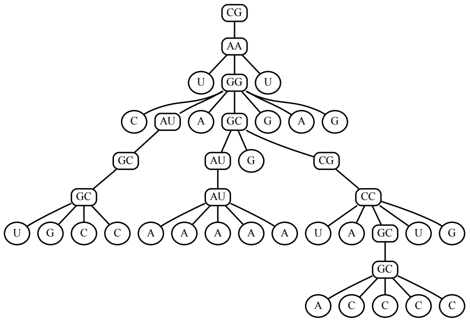

(d) Tree representation of the secondary structure of the modified RNA molecule.

Figure 6: Removing two residues from a multi-branch loop.

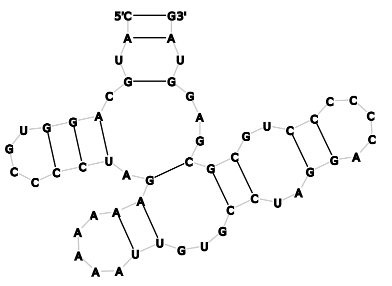

(a) Original RNA molecule

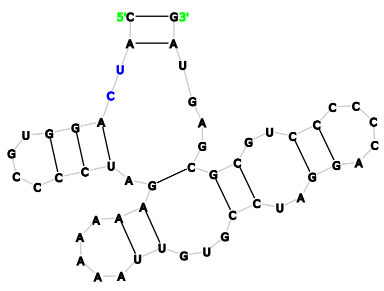

(b) Modified RNA molecule

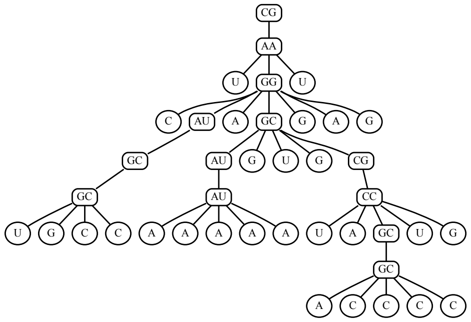

(c) Tree representation of the secondary structure of the original RNA molecule.

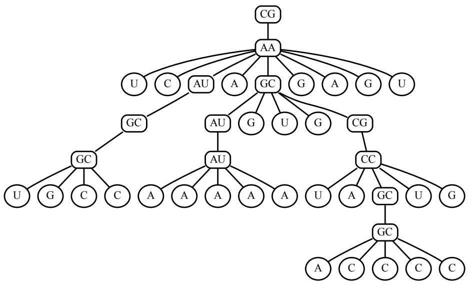

(d) Tree representation of the secondary structure of the modified RNA molecule.

Figure 7: Removing a basepair to extend a multi-branch loop.
